# A hybrid approach for quantifying aortic valve stenosis using impedance cardiography and echocardiography

**DOI:** 10.1186/s12872-015-0155-5

**Published:** 2016-01-22

**Authors:** Yunis Daralammouri, Khubaib Ayoub, Najwan Badrieh, Bernward Lauer

**Affiliations:** Department of Cardiology, Department of Cardiology, Al Najah National University Hospital, Nablus, Palestine; Al-Quds University, Beit Hanina, Palestine; Department of Cardiology, Zentralklinik Bad Berka, Robert-Koch-Allee 9, 99437 Bad Berka, Germany

**Keywords:** Impedance cardiography, Aortic stenosis, Echocardiography, Heart catheterization, Hybrid approach

## Abstract

**Background:**

Impedance cardiography (IC) is a noninvasive modality that utilizes changes in impedance across the thorax to assess hemodynamic parameters, including stroke volume (SV).

This study compared aortic valve area (AVA) as assessed by a hybrid approach of transthoracic echocardiography (TTE) and impedance cardiography (IC) to AVA determined at cardiac catheterization using the Gorlin equation.

**Methods:**

A total of 30 patients with moderate to severe aortic stenosis underwent AVA measurement using two different approaches: using the continuity equation (CE) in a hybrid method combining IC and TTE (AVA = stroke by volume impedance cardiography/trans-aortic-VTI) and using the Gorlin equation. Patient age ranged from 37 to 82 years (mean 48); there were 21 men and 9 women. Twenty-five patients were in sinus rhythm, and five had atrial fibrillation.

**Results:**

There was no statistically significant difference for the mean AVA between the two methods (0.7 ± 0.24 cm^2^ using the Gorlin equation versus 0.7 ± 0.23 cm^2^ using the hybrid approach, *p* = 0.17; r = 0.76, *p* < 0.001). The mean difference was 0.004 cm^2^, and the limits of agreement were −0.33 to 0.37.

**Conclusion:**

The hybrid method using impedance cardiography and TTE is a reasonable, clinically applicable approach to evaluate AVA and has significant correlation to invasive measurement using the Gorlin equation.

## Background

Calcific aortic stenosis is the main heart valve disease in the elderly, leading to focal calcification and thickening of the valve cusps [1] with a prevalence of 1.2 to 1.8 % among patients 65 to 74 years of age and 4.1 to 5.2 % in individuals ≥75 years of age [2]. Doppler echocardiography has gained widespread clinical acceptance as the standard method for evaluating aortic stenosis [3]. Doppler-derived data can be used to calculate the aortic valve area (AVA) by means of the continuity equation (CE). This approach requires measurement of the left ventricular outflow tract (LVOT) diameter, the integral of the pulsed-wave Doppler velocity in the LVOT and the integral of the continuous-wave Doppler velocity across the stenotic valve. Several studies have demonstrated the validity of this approach [4–8]. However, circumstances may arise that can lead to inadequate measurement of the LVOT diameter or LVOT flow. For example, poor acoustic windows or heavy calcification of the aortic valve may hamper the exact measurement of the LVOT diameter, and flow acceleration in the LVOT can lead to an overestimation of the Doppler-derived stroke volume (SV). An alternative method to overcome the difficulties associated with the determination of stroke volume by echocardiography is using thermodilution during right heart catheterization. Simultaneous measurement of the transaortic velocity-time integral (VTI) by Doppler echocardiography allows for the calculation of AVA according to the CE (AVA = SV/transaortic VTI, where SV = stroke volume and VTI = velocity-time integral). Although a valid method for calculating the AVA in aortic stenosis [6, 9], the invasive nature of this procedure limits its routine clinical use. Impedance cardiography (IC) is a feasible, accurate method for the non-invasive measurement of cardiac output in the presence of moderate to severe aortic stenosis [10, 11].

IC applies Ohm’s relationship to the thorax to allow changes in voltage and impedance to be translated into hemodynamic parameters of cardiac function. Because blood is a strong conductor of current compared to the surrounding thoracic tissues, variations in blood flow through the great vessels results in a measurable change in impedance that allows for the calculation of the effective stroke volume [12]. In our hybrid approach, the numerator of the CE (stroke volume) was determined by IC, and the denominator (velocity-time integral of the continuous-wave Doppler through the stenotic valve) was determined by echocardiography.

This study compared the AVA as assessed by the hybrid method using IC and echocardiography-derived data to AVA measured during cardiac catheterization using the Gorlin equation.

## Method

### Study population

The study population consisted of 30 consecutive adult patients undergoing cardiac catheterization with moderate to severe aortic valve stenosis, who were evaluated at our institution. The exclusion criteria included a very poor acoustic window, presence of left-to-right shunting, presence of more than 2+ mitral, aortic or tricuspid regurgitation and the presence of tachyarrhythmias. Rate-controlled atrial fibrillation was not an exclusion criterion. Patients with a known allergy or sensitivity to the sensor gel or adhesive and patients with skin lesions at the sensor placement sites were excluded. The study protocol was approved by the Ethics Committee of Zentralklinik Bad Berka (Germany). Informed consent was obtained from all patients.

### Impedance cardiography

Stroke volume was determined non-invasively using a commercially available system for IC (Task Force Monitor Systems, CNSystems, Graz, Austria). Two electrodes were placed bilaterally on the inferior chest wall in combination with one electrode on the neck. Low-amplitude, high-frequency current was delivered via these surface electrodes, and the transthoracic impedance to this current flow was measured. Changes in transthoracic impedance were measured by four additional surface electrodes: one pair placed bilaterally to the sternum and the second pair placed bilaterally to the abdomen. The stroke volume was calculated on a beat-to-beat basis from the transthoracic impedance signal [13]. An average of stroke volumes during 1 min was calculated.

IC, a complete echocardiographic examination and right heart catheterization were performed within a time period of two hours. Immediately after the IC examination, all of the patients were brought to the cardiac ultrasound laboratory to acquire continuous-wave Doppler spectra of the aortic valve. The heart rate was registered both during the IC and Doppler examinations to ensure that data acquisition was performed approximately at the same heart rate. The ejection fraction was calculated from the echocardiography data.

### Echocardiography

TTE studies were performed and analyzed by the same experienced physician echocardiographer using a commercially available system (Vivid Seven, GE-Vingmed Ultrasound, Horten, Norway). The TTE measurements were performed according to the American Society of Echocardiography guidelines [14]. The velocity-time integral across the aortic valve was measured from the apical 5-chamber view with continuous-wave Doppler. A total of three measurements were performed for each parameter in patients with sinus rhythm, and ten were performed in patients with atrial fibrillation. Calculation of the AVA was performed using the CE: AVA = SV/transaortic VTI, where SV = stroke volume and VTI = velocity-time integral.

### Right and left heart catheterization

Right and left heart catheterization was performed via the right femoral approach. Right heart catheterization was performed using a Swan-Ganz, flow-directed thermodilution catheter and a cardiac output computer. The cardiac output was calculated as the average of three consecutive measurements for patients in sinus rhythm and 10 consecutive measurements for patients in atrial fibrillation. In all patients, the transvalvular gradient was determined by simultaneous measurement of the pressure in the left ventricle and ascending aorta. The AVA was calculated using the Gorlin equation.

### Statistical analyses

The results are expressed as the mean ± SD. The hybrid approach versus catheterization measurements were compared using 2-tailed, paired Student *t*-tests. The correlations and agreements between the hybrid approach and catheterization measurements were assessed by Pearson's correlations and Bland-Altman comparisons, respectively.

## Results

**Fig. 1:**
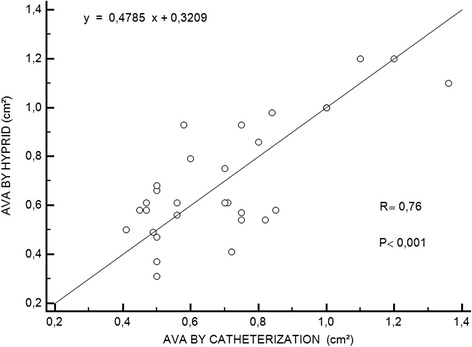
Correlation between AVA determined using catheterization data and using the hybrid approach. Catheterization valve areas were calculated using the standard continuity equation; the AVA derived by the hybrid approach was calculated with the equation AVA = SV/transaortic VTI. (r = .76, *p* < .0001). Abbreviations: AVA; aortic valve area, SV; stroke volume, VTI; velocity-time integral

**Fig. 2:**
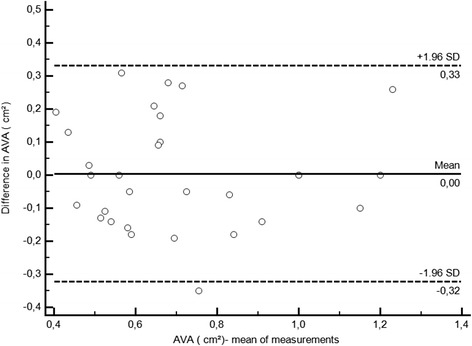
Concordance between the AVA measured by the standard continuity equation and the hybrid approach. The continuous line represents the mean difference, and the dashed lines represent the limits of concordance. Abbreviations: see Fig. 1

**Fig. 3:**
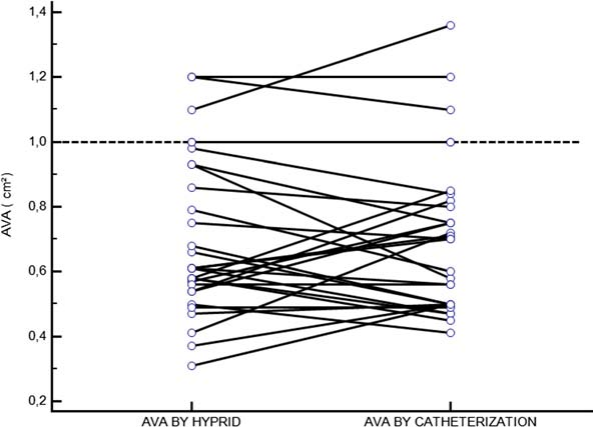
Grading of aortic stenosis severity by AVA using the catheterization or hybrid approach. Abbreviations: see Fig. 1

Thirty patients (21 men, 9 women), ranging from 37 to 82 years (mean 48), were included in the study. Five patients were in rate-controlled atrial fibrillation, and 25 patients were in sinus rhythm. All of the patients had either moderate or severe aortic stenosis, and none of the patients had severe aortic, mitral or tricuspid regurgitation. The ejection fraction ranged from 50 to 70 % (mean 58 ± 12.3 %). There were no statistically significant differences between the mean AVAs as assessed by the two methods (0.70 ± 0.24 cm^2^ by the hybrid method and 0.70 ± 0.23 cm^2^ by the Gorlin formula, *P* = ns). The correlation between the methods was significant (r = 0.76, *p* < 0.01) (Fig. 1). The mean difference was 0.004 cm^2^, and the limits of concordance were −0.32 to 0.33. The Bland-Altman plot is shown in Fig. 2. All of the patients were classified into the same category of severity by both methods (Fig. 3).

## Discussion

Doppler echocardiography has become the preferred method for assessing aortic stenosis in clinical practice because it is non-invasive, widely available and has been extensively studied in the past [4–8]. Calculation of the AVA using the CE requires measurement of the LVOT diameter, velocity-time integral of the blood flow in the LVOT and velocity-time integral of the blood flow across the aortic valve. Inaccurate measurement of one or more of these parameters can lead to significant errors in the determination of the AVA. Poor acoustic windows and heavy calcification of the aortic valve are the predominant reasons for inadequate determination of the LVOT diameter. The continuity equation further amplifies this error by using the square of the LVOT diameter. Flow acceleration in the LVOT is another source of error. It can lead to the overestimation of the numerator in the CE.

To overcome the difficulties associated with the standard CE, alternative methods have been discussed. These methods include using transesophageal echocardiography for direct planimetry of the AVA [15–18] or standard CE using a transesophageal approach [19, 20] as well as CMR using planimetry of the AVA or velocity-encoded CMR to calculate the AVA according to the standard CE [21].

However, none of these methods has been shown to be superior, and each method has its limitations. Transesophageal echocardiography is semi-invasive and not feasible in all patients [22]. Additionally, severe valvular calcification may prevent clear visualization of the orifice area [20]. Heavy calcification and turbulent flow across the aortic valve can also lead to difficulties in edge discrimination due to signal void during CMR imaging [23, 24]. Clinical experience in assessing aortic stenosis by velocity-encoded CMR is limited, and until real-time flow recording becomes available, velocity aliasing might be a source of inefficiency in this method [21]. Additionally, CMR is time consuming and costly and certainly will not replace Doppler echocardiography in everyday clinical practice.

A hybrid approach consisting of stroke volume assessed by thermodilution during right heart catheterization and simultaneous measurement of the transaortic velocity-time integral assessed by Doppler echocardiography is a good alternative method [6, 9]. The validity of this method could be demonstrated by comparing measurements during left heart catheterization using the Gorlin equation. However, right heart catheterization is an invasive procedure with inherent risks.

In this study, we have demonstrated that there was no significant difference between mean aortic valve area (AVA) using the hybrid IC-Doppler approach in the continuity equation and the invasive measures using Gorlin equation with good significant correlation between both methods.

Goli et al. have used the hybrid IC-Doppler approach and found that AVA using this method is not significantly different from the invasive measures [10]. Instead of using continuity equation, they used IC to calculate cardiac output and Doppler echocardiography to figure out trans-valvular pressure gradient then calculated AVA using Gorlin equation. On the other hand, they performed AVA calculation using continuity equation (CE) with all the data obtained from Doppler echocardiography only and showed good AVA correlation with the invasive measures but less than the hybrid IC-Doppler approach with Gorlin equation.

Combining measurements of stroke volume by IC with measurements of velocity-time integral across the aortic valve by echocardiography in a hybrid approach offers several advantages. First, this method is an applicable, feasible, accurate, efficient, cheap and non-invasive method for measuring SV that can be frequently used in aortic stenosis follow-up and in patients with a poor acoustic window or inaccurate LVOT measurement. Second, it bypasses the difficulties associated with the determination of LVOT diameter and LVOT flow by echocardiography.

Moreover, the use of hybrid IC-Doppler derived data to calculate AVA using continuity equation (CE) instead of Gorlin equation as done by Goli et el. [10] may be more accurate as many studies showed that Gorlin equation tends to over-estimate the valve area and it may reflect the anatomical rather than functional area [25, 26].

Most importantly, all of the patients were classified into the same category of severity by both methods, as shown by (Fig. 3). The results showed that all patients who have AVA less than 1 cm^2^ using hybrid IC-Doppler method also have AVA less than 1 cm^2^ by the invasive measures (Gorlin equation) and this applies also to those with AVA more than 1 cm^2^. This means that even if there is a difference between hybrid IC-Doppler and invasive measures estimation of the AVA, the grade of the stenosis will not differ and this will make better justifications of the decisions made based on this estimation, like doing valve replacement for example.

The IC-Doppler hybrid method has limited value in severe mitral regurgitation, severe tricuspid regurgitation, severe aortic regurgitation and significant left-to-right shunting [27–29]. Moreover, Impedance cardiography should not be used for patients with severely abnormal cardiac or thoracic anatomy [30]. There are clinical conditions associated with underestimated measurements of the cardiac output and stroke volume in comparison with the invasive thermodulation method. These conditions includes hyperdynamic status (e.g. Septic shock), tachycardia and cardiac arrhythmia [31]. The estimation of Stroke volume by impedance cardiography can also be inaccurate, when there is a considerable degree of arterial hypertension, in case of short and tall population (Patients height less than 120 cm or over 230 cm) and in thin or obese patients (weight is less 30 kg or over 155 kg) [32].

The results of our non-invasive IC-derived hybrid approach may be equally used instead of left heart catheterization-derived to determine the AVA in aortic valve stenosis. Comparing both methods by Bland-Altman analysis showed a mean difference close to zero, a range within two standard deviations and very similar limits of agreement.

### Limitations

There are some limitations of the method used (IC-Doppler hybrid). Impedance cardiography assumes that the chest is a symmetrical cylinder, so this method cannot be applied in patients with thoracic anatomic abnormalities and those with height or weight extremes as mentioned earlier [30, 32]. However, these patients represent a minority of patients in which we need to use invasive measures.

Another minority of patients that hybrid IC-Doppler may underestimate cardiac output and thus affect AVA calculation like in tachycardia, arrhythmias and conditions with hyperdynamic status like in septic shock, but other non-invasive methods also may be less accurate in these settings and the patients may be hemodynamically unstable to undergo AVA estimation. Additionally, the IC-Doppler hybrid method has limited value in severe valvular diseases including severe mitral, tricuspid and aortic regurgitation and significant left-to-right shunting.

Secondly, the impedance cardiography (IC) was done once to each patient which may raise the query about reproducibility and consistency of this method. However, IC effectiveness in estimation of cardiac output (CO) was proved to be reproducible and comparable or superior to other methods of CO estimation in previous studies [13].

Thirdly, the absence of a control group may limit the validity of the results but there is no control group in most of the studies of this type as there is an ethical and practical problems in doing invasive risky procedures like heart catheterization in normal people.

Lastly, additional studies with a greater number of patients, including those with a low ejection fraction or prosthetic aortic valve must be performed in order to explore the utility of widespread use of this new method.

## Conclusions

A hybrid approach using impedance cardiography and echocardiography provides an applicable, feasible, accurate, efficient, cheap and non-invasive method for measuring SV that can be frequently used in aortic stenosis follow-up and in patients with a poor acoustic window or inaccurate LVOT measurement because it bypasses the difficulties associated with determining the LVOT diameter by echocardiography.

## Abbreviations

AVA: Aortic valve area
IC: Impedance cardiography
TTE: Transthoracic echocardiography
CE: Continuity equation
LVOT: Left ventricular outflow tract
VTI: Velocity-time integral
CMR: Cardiovascular magnetic resonance imaging

## References

[1] Kaden JJ, Vocke DC, Fischer CS, Grobholz R, Brueckmann M, Vahl CF, et al. Expression and activity of matrix metalloproteinase-2 in calcific aortic stenosis. Z Kardiol. 2004;93:124–30. 10.1007/s00392-004-1021-0 14963678

[2] Nkomo VT, Gardin JM, Skelton TN, Gottdiener JS, Scott CG, Enriquez-Sarano M (2006). Burden of valvular heart diseases: a population-based study. Lancet..

[3] Baumgartner H, Hung J, Bermejo J, Chambers JB, Evangelista A, Griffin BP (2009). Echocardiographic assessment of valve stenosis: EAE/ASE recommendations for clinical practice. J Am Soc Echocardiogr.

[4] Zoghbi WA, Farmer KL, Soto JG, Nelson JG, Quinones MA (1986). Accurate noninvasive quantification of stenotic aortic valve area by Doppler echocardiography. Circulation.

[5] Oh JK, Taliercio CP, Holmes Jr DR, Reeder GS, Bailey KR, Seward JB, et al. Prediction of the severity of aortic stenosis by Doppler aortic valve area determination: prospective Doppler-catheterization correlation in 100 patients. J Am Coll Cardiol. 1988;11:1227–34. 10.1016/0735-1097(88)90286-0 3366997

[6] Otto CM, Pearlman AS, Comess KA, Reamer RP, Janko CL, Huntsman LL. Determination of the stenotic aortic valve area in adults using Doppler echocardiography. J Am Coll Cardiol. 1986;7:509–17. 10.1016/s0735-1097(86)80460-0 3950230

[7] Skjaerpe T, Hegrenaes L, Hatle L (1985). Noninvasive estimation of valve area in patients with aortic stenosis by Doppler ultrasound and two-dimensional echocardiography. Circulation.

[8] Currie PJ, Hagler DJ, Seward JB, Reeder GS, Fyfe DA, Bove AA (1986). TajikAJ Instantaneous pressure gradient: a simultaneous Doppler and dual catheter correlative study. J Am Coll Cardiol.

[9] Warth DC, Stewart WJ, Block PC (1984). Weyman AE. A new method to calculate aortic valve area without left heart catheterization. Circulation.

[10] Goli VD, Teague SM, Prasad R, Harvey J, Voyles WF, Olson EG, et al. Noninvasive evaluation of aortic stenosis severity utilizing Doppler ultrasound and bioimpedance. Journal of the American College of Cardiology. 1988;11(1):66–71. 10.1016/0735-1097(88)90168-4 3335708

[11] Daralammouri Y, Gayed M, Geller JC, Lauer B. Non-Invasive Cardiac Output Assessment by Impedance Cardiography in Patients with Moderate to Severe Aortic valve Stenosis: Comparitive Study with Thermodilution Method. Clin Res Cardiol 100, Suppl 1, April 2011(Abstract P1012)

[12] Rosenberg P, Yancy CW (2000). Noninvasive assessment of hemodynamics: an emphasis on bioimpedance cardiography. Curr Opin Cardiol.

[13] Ovsyshcher I, Furman S (1993). Impedance cardiography for cardiac output estimation in pacemaker patients: review of the literature. Pacing Clin Electrophysiol.

[14] Quiñones MA, Otto CM, Stoddard M, Waggoner A, Zoghbi W. Doppler Quantification Task Force of the Nomenclature and Standards Committee of the American Society of E: Recommendations for quantification of Doppler echocardiography: a report from the Doppler Quantification Task Force of the Nomenclature and Standards Committee of the American Society of Echocardiography. J Am Soc Echocardiogr. 2002;15:167-84. doi:10.1067/mje.2002.120202. 10.1067/mje.2002.120202 11836492

[15] Kim KS, Maxted W, Nanda NC, Coggins K, Roychoudhry D, Espinal M (1997). Comparison of multiplane and biplane transesophageal echocardiography in the assessment of aortic stenosis. Am J Cardiol.

[16] Tardif JC, Rodrigues AG, Hardy JF, Leclerc Y, Petitclerc R, Mongrain R, et al. Simultaneous determination of aortic valve area by the Gorlin formula and by transesophageal echocardiography under different transvalvular flow conditions. Evidence that anatomic aortic valve area does not change with variations in flow in aortic stenosis. J Am Coll Cardiol. 1997;29:1296–302. 10.1016/s0735-1097(97)00060-0 9137227

[17] Hoffmann R, Flachskampf FA, Hanrath P (1993). Planimetry of orifice area in aortic stenosis using multiplane transesophageal echocardiography. J Am Coll Cardiol.

[18] Tribouilloy C, Shen WF, Peltier M, Mirode A, Rey JL, Lesbre JP (1994). Quantitation of aortic valve area in aortic stenosis with multiplane transesophageal echocardiography: comparison with monoplane transesophageal approach. Am Heart J.

[19] Blumberg FC, Pfeifer M, Holmer SR, Kromer EP, Riegger GA, Elsner D (1997). Transgastric Doppler echocardiographic assessment of the severity of aortic stenosis using multiplane transesophageal echocardiography. Am J Cardiol.

[20] Blumberg FC, Pfeifer M, Holmer SR, Kromer EP, Riegger GA, Elsner D (1998). Quantification of aortic stenosis in mechanically ventilated patients using multiplane transesophageal Doppler echocardiography. Chest.

[21] Caruthers SD, Lin SJ, Brown P, Watkins MP, Williams TA, Lehr KA, et al. Practical value of cardiac magnetic resonance imaging for clinical quantification of aortic valve stenosis: comparison with echocardiography. Circulation. 2003;108:2236–43. 10.1161/01.CIR.0000095268.47282.A1 14568899

[22] Tribouilloy C, Shen WF, Peltier M, Mirode A, Rey JL, Lesbre JP (1994). Quantitation of aortic valve area in aortic stenosis with multiplane transesophageal echocardiography: comparison with monoplane transesophageal approach. Am Heart J.

[23] John AS, Dill T, Brandt RR, Rau M, Ricken W, Bachmann G, et al. Magnetic resonance to assess the aortic valve area in aortic stenosis: how does it compare to current diagnostic standards? J Am Coll Cardiol. 2003;42:519–26. 10.1016/s0735-1097(03)00707-1 12906983

[24] Kupfahl C, Honold M, Meinhardt G, Vogelsberg H, Wagner A, Mahrholdt H, et al. Evaluation of aortic stenosis by cardiovascular magnetic resonance imaging: comparison with established routine clinical techniques. Heart. 2004;90:893–901. 10.1136/hrt.2003.022376 PMC1768383 15253962

[25] Rudolph V, Hartrumpf M, Schelenz C, Hüttemann E, Albes JM, Reinhart K, et al. Comparison of continuity equation and Gorlin formula effective orifice area of aortic valve prostheses: an intraoperative study. European Journal of Anaesthesiology (EJA). 2002;19:11.

[26] Chambers JB, Sprigings DC, Cochrane T, Allen J, Morris R, Black MM, et al. Continuity equation and Gorlin formula compared with directly observed orifice area in native and prosthetic aortic valves. Br Heart J. 1992;67(2):193–9. 10.1136/hrt.67.2.193 PMC1024754 1540443

[27] Campos PC, D’Cruz I (2004). Functional mitral regurgitation in decompen-sated heart failure: combined bio-impedance and 2D echocardiography follow-up monitoring. Echocardiography..

[28] Boerboom LE, Kinney TE, Olinger GN, Hoffmann RG (1993). Validity of cardiac output measurement by the thermodilution method in the presence of acute tricuspid regurgitation. J Thorac Cardiovasc Surg.

[29] Woo MA, Hamilton M, Stevenson LW, Vredevoe DL (1991). Comparison of thermodilution and transthoracic electrical bioimpedance cardiac outputs. Heart Lung.

[30] Summers RL, Shoemaker WC, Peacock WF, Ander DS, Coleman TG (2003). Bench to bedside: electrophysiologic and clinical principles of noninvasive hemodynamic monitoring using impedance cardiography. Acad Emerg Med.

[31] Newman DG, Callister R (1999). The non-invasive assessment of stroke volume and cardiac output by impedance cardiography: a review. Aviat Space Environ Med.

[32] Sodolski T, Kutarski A (2007). Impedance cardiography: A valuable method of evaluating haemodynamic parameters. Cardiol J.

